# The future of HIV testing in eastern and southern Africa: Broader scope, targeted services

**DOI:** 10.1371/journal.pmed.1004182

**Published:** 2023-03-14

**Authors:** Anna Grimsrud, Lynne Wilkinson, Peter Ehrenkranz, Stephanie Behel, Thato Chidarikire, Tina Chisenga, Rachel Golin, Cheryl Case Johnson, Maureen Milanga, Obinna Onyekwena, Maaya Sundaram, Vincent Wong, Rachel Baggaley

**Affiliations:** 1 IAS–International AIDS Society, Cape Town, Western Cape, South Africa; 2 University of Cape Town, Cape Town, South Africa; 3 Bill & Melinda Gates Foundation, Seattle, Washington, United States of America; 4 Centres for Disease Control and Prevention, Atlanta, Georgia, United States of America; 5 South Africa National Department of Health, Pretoria, Gauteng, South Africa; 6 Zambia Ministry of Health, Lusaka, Zambia; 7 Office of the Global AIDS Coordinator and Health Diplomacy, Washington DC, United States of America; 8 United States Agency for International Development, Washington DC, United States of America; 9 World Health Organization, Geneva, Switzerland; 10 Health GAP, Nairobi, Kenya; 11 The Global Fund, Geneva, Switzerland; N/A, UNITED KINGDOM

## Abstract

In this Policy Forum, Anna Grimsrud and colleagues discuss the future of HIV testing in eastern and southern Africa, using insights gleaned from a 2021 expert consultation.

**Summary points**

- Scale-up of HIV testing services (HTS), primarily through routine offer of HIV testing in health services, has led to an increase in the proportion of people with HIV who know their status and are accessing HIV treatment.
- In eastern and southern Africa (ESA), home to more than half of people living with HIV globally, many countries are close to reaching global targets for HIV treatment and viral suppression, with slower progress towards the global target that 95% of people should know their HIV status. Given this, it is critical to update the approach to HIV testing to reflect changes in the HIV epidemic, the response to it, and to acknowledge ongoing resource constraints.
- An expert consultation series defined this updated approach as a shift to “broader scope, targeted services.” Over the next decade, HTS in ESA should implement a status-neutral approach that maintains core testing services to reach the greatest number of people with HIV not on treatment, while broadening the scope to support linkage to appropriate prevention and treatment. It is important that HTS programs use a strategic mix of modalities focused on people most likely to have undiagnosed HIV, those who are not on ART, and people who are more vulnerable to HIV acquisition.
- Ten key themes for the future of HTS were articulated. The most critical are: promote a status-neutral approach to HTS; realize the potential of HIV self-testing (HIVST); prioritize facility-based HTS; reframe retesting among those previously diagnosed but not currently on antiretroviral therapy (ART) as an opportunity; and involve and invest in community leadership and community-led monitoring (CLM) to ensure HTS meets the needs and preferences of clients.
- The country-specific epidemiological context must inform the focus and mix of testing approaches. Testing programs should acknowledge regional transmission dynamics including that the majority of new infections are acquired from people living with HIV for longer than a year, with transmission driven by many who transmit to a few, rather than by a few who transmit to many.
- HTS programs should not reduce the volume of HIV testing. Rather HTS programs should broaden the scope of testing to encapsulate both prevention and treatment objectives and prioritize services to the people at the highest risk of HIV.

## Introduction

In the 40 years since AIDS was first reported, many countries in eastern and southern Africa (ESA), the region most affected by HIV, have made substantial progress towards the global 2025 HIV testing target of 95% of people with HIV being diagnosed [1]. Knowledge of HIV status increased from 5% to 86% in eastern Africa and 9% to 90% in southern Africa between 2000 and 2020. [2]. Concurrently, there was a decrease in the median time to diagnosis (11 and 7.7 years to 2.0 and 1.5 years), HIV positivity (11% and 15% to 2.5% and 5.5%), and the proportion of first-time diagnoses among all positive tests (91% and 87% to 41% and 39%) [2]. While the decrease in time to diagnosis is a remarkable achievement, the decrease in positivity and proportion of first-time diagnoses highlight the need for HTS to evolve to meet the needs of the HIV response. Continuous optimization of effective strategies, innovation, and monitoring are needed to accelerate the decline in HIV incidence.

## Defining the future of HIV testing through an expert consultation series

In 2021, International AIDS Society (IAS), the World Health Organization (WHO), and the Bill & Melinda Gates Foundation (BMGF) convened an expert consultation, “*The future of HIV testing*: *Beyond reaching the first 95 in sub-Saharan Africa*”, to define a vision for HIV testing services (HTS) for the next decade in the context of an evolving epidemic and response. While the consultation title referred to sub-Saharan Africa, the discussion was more specifically focused on high-HIV burden countries in ESA. The expert consultation, a virtual three-part series, included 78 stakeholders who were invited based on their experience in HTS and who live or work in 11 ESA countries. Sessions 1 (“Past success, present priorities and future opportunities”) and 2 (“The different, and sometimes competing, roles of HIV testing”) included presentations and discussions. Session 3 was a response to the first 2 sessions and involved breakout groups to facilitate inputs from all participants. The consultation agenda is available as S1 Appendix and details of the participants in S2 Appendix. Participants articulated a vision of the future of HIV testing in the region and 10 supporting key themes (Table 1). In this paper, we present this vision along with key themes.

**Table 1:** Key themes identified during the expert consultation series on the future of HIV testing.

Theme	Role in the future of HIV testing	Shift toward	Shift away from	Example(s)
1. Broaden global understanding of HTS as a status-neutral approach requiring linkage to and engagement in prevention and treatment services	Recognize importance of HTS beyond HIV case finding Emphasize importance of linkage to and engagement in prevention and treatment services, prioritizing people with vulnerabilities	• Emphasizing value of linking people with a negative test result and with ongoing risk of acquiring HIV to appropriate prevention services (including PrEP and VMMC) • Maximizing absolute number of HIV diagnoses • Reducing cost of HIV test kits by increasing market diversity and monitoring country purchase and utilization of consumables for which price reductions have been negotiated by global stakeholders[3] • Ensuring HTS providers own responsibility for linkage to prevention and/or treatment services with providers of those services • Evaluating HTS linkage and engagement success against prevention and treatment targets	• Yield/positivity and case identification as the sole or primary indicators of HTS program success • Siloed and limited prevention and treatment linkage and engagement mechanisms	• Providing integrated PrEP services to women and girls who test negative within maternal and child health services in Kenya [4] • Supporting young men who are at increased risk of HIV to test and link to services using social media and peer navigation in Nigeria [5])
2. Realize the potential of HIVST	Expand and scale HIVST to reach undertested populations within routine and specialized testing approaches and to support uptake and sustained use of HIV prevention services	• Increasing access through greater use of HIVST within core (facility-based) and prioritized testing approaches (index testing, social-network testing, secondary distribution and partner approaches and other focused private sector and community testing approaches) • Addressing age of consent barriers that limit use of HIVST by adolescents • Expanding effective and acceptable HIVST distribution approaches to reach undertested populations and those who would benefit from simplified access to regular testing • Increasing population-level HIVST literacy towards broader self-testing and self-care literacy • Increasing use for status monitoring among PrEP users to facilitate differentiated PrEP service delivery models • Invest in simplified data collection for HIVST such as triangulation methods	• Narrow distribution of HIVST through a few distribution channels • Distribution focused only on reaching those not currently reached with HTS • Complex and costly data collection about individual users to establish linkage and other outcomes of HIVST • Small pilots with restricted reach among limited populations	• Utilizing HIVST within facilities to increase testing among men and youth, attending the service or accompanying others attending the service, in Malawi [6] • Increasing the number of people diagnosed with HIV with an increase in distribution of HIVST kits in Côte d’Ivoire [7] • Reaching populations less likely to test with peer-delivered messaging and HIVST test kits in Zimbabwe [8]
3. Continue prioritizing facility HTS	Recognize that facility-based HTS is critical for people presenting at health facilities and is resource efficient It increases the reach of index and social network testing for those diagnosed with HIV Based on context, other priority approaches should complement facility-based HTS	• Recognizing that each service entry point is an important testing entry point and reinforcing the availability of HTS on request at all health facilities [9] • Routinely offer HIV testing (rapid or HIVST) at all entry points with suboptimal coverage (e.g., STI and contraceptive services) • Evaluating facility-based HIV testing through a lens of treatment-adjusted prevalence [10]	• Rationing of and reducing facility-based HTS in outpatient settings • Risk-related screening-out tools that likely lead to missing cases, may stigmatize HTS and undermine treatment and prevention goals	• Ensuring access to HIV testing at all health facilities, even during COVID-19, such as in Zambia [11] • Using treatment-adjusted prevalence as a benchmark for anticipating positivity within a testing program [10]
4. Scale use of targeted testing approaches to reach untested individuals	Increase investment in and implementation of targeted community-based testing approaches such as index and social-network testing depending on gaps, epidemiology, and context	• Defining acceptable approaches for key populations and other underserved populations, including social-network and partner services, HIVST, etc. • Involving communities in designing, delivering, and monitoring community-led HTS • Increasing use of virtual platforms as part of empowerment and engagement strategies • Using all data available to inform targeted testing	• General, “all population” community-based testing campaigns • Efforts focused on adolescents and young people without sufficient epidemiological rationale (e.g., high incidence) • Low volume HTS investments covering small numbers of a key population group	• Increasing HIV testing among key populations through peer-to-peer community-based distribution of HIVST and among sexual partners of pregnant women through secondary distribution of HIVST in Uganda [12] • Using peer recruitment strategies to engage those at increased vulnerability in HIV prevention and treatment services in Côte d’Ivoire [13]
5. Reframe retesting among those previously diagnosed as an opportunity for essential (re)engagement	Understand and accept that a subset of people who know they have HIV will attend HTS for retesting. Use HTS as an opportunity for those not successfully linked to ART or who have disengaged from ART to (re)start treatment.	• Recognizing that retesting and “diagnosis confirmation” is a legitimate and effective linkage strategy for ART engagement • Accepting and productively engaging with individuals who use HTS as a means of returning to ART care [14] • Strengthening health information systems to identify individuals previously diagnosed who are re-engaging in ART care through retesting	• Presuming that those testing positive are newly diagnosed • Considering retesting (apart from verification testing) among those who were previously diagnosed as always wasteful • Not addressing underlying reasons for retesting among those previously diagnosed [15]	• Reviewing routine data to see the proportion of those initiating treatment who are non-naïve such as in South Africa [16] • Prior exposure to ART among adult patients presenting for HIV treatment initiation or re-initiation in sub-Saharan Africa: a systematic review [14]
6. Involve communities and invest in CLM	Increase community engagement through leadership and participation in HTS design, monitoring and adaptation of HTS to ensure services meet the needs and preferences of people using the service and that service delivery is adapted to reach those least likely to access care	• Recommitting to broader community leadership, engagement, and participation in policy development, program design, and demand creation for HTS • Increasing financial, technical expertise, and political buy-in for sustained CLM of HTS • Advocating for HTS quality-related indicators to be part of CLM for both first time and repeat testers (including exploring previous ART experience)	• Neglecting the benefits of community leadership • CLM being limited to HIV treatment and facility-based services	• Using CLM data to co-create solutions to increase HIV testing such as among key populations and young people [17] • Involving trusted community leaders in HTS such as traditional healers offering point-of-care HIV tests in Uganda [18]
7. Integrate person-centered HTS into primary healthcare services that prevent, diagnose, and treat a full range of health conditions	Promote integration of HTS into broader clinical services and integration of complementary clinical services into HTS, encourage multi-condition screening to foster person-centered healthcare services [19]	• Offering HIV testing as part of multi-condition screening including use of dual/combination tests based on context and population appropriateness [20] • Enhancing bi-directional HTS and other clinical services integration	• Siloed HTS programs and funding not supporting integrated service provision or multi-condition screening • Limited multi-disease and multi-sectoral engagement with service delivery and priority setting	• Integrating HIV testing and treatment within chronic disease screening and treatment in Kenya and Uganda [21]
8. Expand use of virtual interventions and digital tools to support HTS	Scale digital tool use to increase access, reach, acceptability, and long-term cost–effectiveness of HTS	• Building HTS capacity in target populations with members who successfully engage social networks through virtual platforms • Evaluating and scaling successful virtual provider-patient approaches and program adaptations introduced during COVID-19 [22]		• Supporting HIVST via digital platforms to expand reach and uptake [23] • Using virtual interventions including social media, live chats, short message services, and apps, to bolster HTS [24]
9. Improve community prevention and treatment literacy, including U = U messaging	Provide population- and provider-level HIV prevention and treatment literacy, including the preventive benefits of ART (“U = U” messaging) [25]	• Recognizing that population-level stigma reduction remains critical to reaching those underserved [26] • Investing in multi-stakeholder buy-in and delivery of population-level country-specific HIV epidemic literacy with a focus on locally adapted U = U related education and messaging [27]	• Focusing on HIV literacy for only those with HIV	• Using peer-delivered U = U messaging to increase uptake of testing among men in South Africa [27]
10. Regularly update strategic mix of differentiated HTS	Train/support stakeholders at all levels to regularly review, innovate, and optimize the implemented mix of HTS to address current local epidemiological needs	• Increasing the capacity of national and subnational staff to optimize HTS programs • Increase reliability, availability, and public access to appropriate data [28]		• Utilizing a mix of person-centered differentiated HIV testing approaches to reach key populations in Malawi [28] • Identifying where to offer mobile community HIV testing based on site-level data and information from key informants in South Africa [29]

ART, antiretroviral therapy; CLM, community-led monitoring; COVID-19, Coronavirus Disease 2019; HIVST, HIV self-testing; HTS, HIV testing service; PrEP, pre-exposure prophylaxis; STI, sexually transmitted infection; VMMC, voluntary medical male circumcision.

### A vision for HIV testing services in ESA for the next decade: Broader scope, targeted services

Our vision for HTS in ESA for the next decade involves an important shift, where the scope of HTS is broadened towards “status-neutral” testing that actively supports linkage to and engagement in prevention or treatment programs [30]. The shift will increase HTS volume while also targeting services towards the people who are most likely to be at risk of acquiring HIV. It also emphasizes continual country-led and evidence-based adaptation of HTS to an evolving HIV epidemic, with a commitment to human rights and community engagement. Metrics of success will extend beyond HIV positivity. Countries could collectively adapt this vision to revise national HTS policies, program planning, and implementation, including related targets and indicators.

## Broaden understanding of HTS as a status-neutral approach

### Background

Knowledge of HIV status is only one aspect of HTS. To maximize individual and public health benefits, it is critically important that HTS actively links individuals to prevention and treatment services and support them to remain engaged in care; those who test negative need to be linked, with support, to prevention services. Previously, when there was limited access to treatment and prevention interventions, HTS emphasized “know your status.” As treatment became more available, there was increased emphasis on the HIV positivity rate and rapid linkage to ART to achieve viral load suppression, to improve the individual’s health, and to decrease HIV transmission to others. HTS was integral to a “treat all” approach that focused on case finding and linkage to treatment services.

### Importance to the future of HIV testing

**Fig 1:**
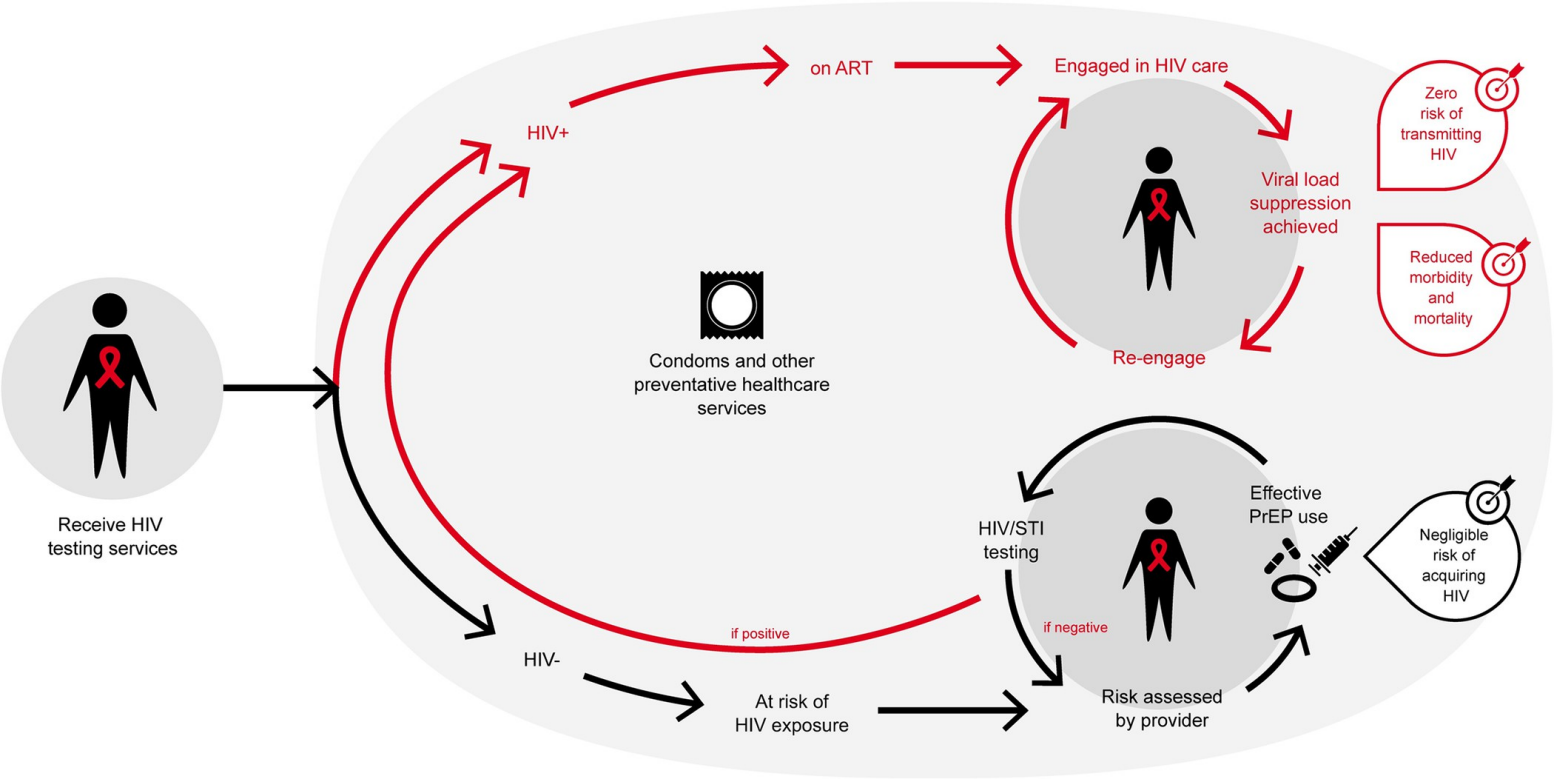
An example of a status-neutral approach to HIV testing with linkage to PrEP [31]. Legend: HIV testing services result in an action—either the red arrows for someone who is living with HIV or the black arrows for the person who tests HIV negative. HIV testing services are therefore status-neutral, with benefits and an action regardless of the test outcomes. The person on the left receives HIV testing services. If they test positive for HIV, they follow the red arrow up and are linked to and initiated on ART. They continue to follow the red arrows and are engaged in HIV care and achieve viral suppression. They may interrupt treatment and need to be re-engaged in care. By achieving and sustaining viral suppression, they have zero risk of transmitting HIV and reduced morbidity and mortality. If the person on the left receives HIV testing services and tests negative for HIV, they will follow the black arrows along the bottom. If they are at ongoing risk of HIV exposure, they should have their risk assessed by a provider. They may need to be provided with PrEP and supported to use PrEP effectively to have negligible risk of HIV acquisition. While on PrEP, they should also be provided with frequent HIV and STI testing. If they do acquire HIV, they then follow the red arrow left and to the top to be provided with services for a person living with HIV. Both people living with HIV and those without HIV should be provided with condoms and other preventive healthcare services. ART, antiretroviral therapy; HIV+, person living with HIV who tests positive; HIV-, person who tests negative for HIV; HIV/STI, HIV and sexually transmitted infection, PrEP, pre-exposure prophylaxis.

Biomedical prevention options continue to expand beyond the cornerstones of condoms, voluntary medical male circumcision (VMMC), and prevention services for key populations, to include an increasing range of pre-exposure prophylaxis (PrEP) options. A parallel shift in HTS delivery—to status-neutral testing—is required [30]. This approach is intended to broaden HTS beyond case-finding, to serve populations at ongoing risk of HIV acquisition by expanding access to HTS and actively linking clients to prevention services (Fig 1) [31]. It also supports early identification of new cases through partner, index, and social-network testing, and linkage to and support for sustained engagement in treatment services for those who test positive [32].

Shifting HTS to a status-neutral approach requires buy-in from stakeholders, adequate funding, and revised targets and metrics including new HTS indicators for linkage to prevention and retesting coverage as defined in the WHO 2022 guidelines on person-centered HIV strategic information [33]. Increased referral from HTS should result in increased uptake and more effective engagement in prevention services, requiring additional resources. Measures of HTS success should include both prevention (condom programming, PrEP, needle and syringe programming (NSP), opioid agonist maintenance therapy (OAMT), VMMC, and PMTCT) and treatment (ART initiations, re-engagement in ART).

Broadening the scope of HTS will increase HTS volume and therefore require strategic financing approaches. Global stakeholders will need to prioritize and facilitate reduced costs for professional use HIV rapid test kits while continuing to reduce HIVST costs through market diversification and development, promotion of pooled purchasing power, support for country tender negotiation processes, and direct manufacturer price negotiation strategies. The recent announcement of US$ 1 HIV self-tests is one example of innovation in the funding space [30]. Maximizing the use of dual HIV/syphilis rapid testing is another potentially cost-effective strategy in antenatal care (ANC) settings [34].

## Realize the potential of HIV self-testing

### Background

HIVST is an important tool for increasing access to and uptake of HTS. HIVST enables people to test when and where they prefer and to initiate linkage and engagement in treatment and/or prevention services. It is a highly acceptable and effective way to reach people who do not test through other HTS delivery options or who need more frequent testing due to ongoing HIV risk [35]. Since 2017, the number of countries with policies supportive of HIVST and where HIVST kits are distributed has increased significantly [36], but there are still many countries where HIVST is not supported. More work on national policy adoption and resources necessary to realize the full potential of HIVST is needed. Tracking of each HIVST kit adds complexity to the process, particularly with secondary distribution models, and compromises anonymity. It may be more effective to monitor kit distribution through public sector tracking models or private sector sales volumes, identifying the groups being reached via self-report during population-based surveys, and using routine HTS data to triangulate HIVST impact.

### Importance to the future of HIV testing

HIVST is not an alternative to conventional testing but an additional testing option for individuals who have not tested or who are unlikely to test through other approaches [37]. HIVST can play a critical role within HIV testing modalities—including as a highly sensitive “screening-in tool” at health facilities. This option can reduce pressure on the health workforce while increasing testing opportunities through other entry points such as outpatient [38], contraception, and/or sexually transmitted infection (STI) services. HIVST also increases community- and workplace-based access for specific populations including adolescents, key populations, and men [39]. Social and virtual networks remain underutilized for distribution of HIVST kits and may benefit from improved messaging for specific populations about the benefits of HIVST. HIVST is also useful for increasing uptake of PrEP by reducing the need to return to health facilities for HTS and thus supporting differentiated PrEP service delivery models [40]. Ultimately, HIVST could play an important role in rebranding HIV testing as a regular part of self-care for certain populations and geographical settings, similar to the role of self-testing for Coronavirus Disease 2019 (COVID-19).

## Continue prioritizing facility-based HTS

### Background

Quality facility-based HTS is the backbone of an HTS program. However, with declining resources for testing and a focus on the positivity rate, countries have been pushed to reduce lower-“yield” HIV testing approaches, trading case-finding volume for testing positivity. Outpatient departments have seen the largest reductions in HIV testing. COVID-19 provided a real-world case study of the impact of large reductions in delivery of facility-based HTS: In 2020, fewer people, particularly men, were tested for HIV, resulting in fewer ART initiations [41–43]. Reducing facility-based HTS may also reduce opportunities to identity clients with advanced HIV disease who present only when acutely ill and who will benefit from the earliest possible diagnosis and treatment.

The diffuse nature of HIV transmission in ESA is characterized by a large number of people at a low risk of acquiring HIV, making provider-initiated testing and counseling (PITC) particularly important [44]. Recent analysis highlights transmission in small, diffuse clusters driven by “many who transmit to a few, rather than by a few who transmit to many” [45]. Further, more than three-quarters of new HIV infections are acquired from people who have been living with HIV for more than a year [46], counter to a previous assumption that identification of new infections to prevent transmission should be the primary focus of prevention programming.

### Importance to the future of HIV testing

Facility-based HTS is not requiring testing of every person but offering HTS to those presenting to health services with conditions or preventive health needs that may indicate a need for HTS. There is no evidence that the “worried well” represent a significant proportion of people seeking testing in facilities, and screening tools used to reduce HIV testing volumes within health facilities for adults are not often validated and have been shown to miss people who would have been otherwise tested [47]. Rather than limiting the routine offer of testing, a more evidence-based approach would be to align the volume of clinic-based HIV testing offered across clinical services (e.g., outpatient, ANC, TB, or STI) informed by epidemiological and program data [11]. In much of ESA, testing in ANC, TB, STI, and contraception outpatient services should be prioritized. The success of these efforts could then be measured with treatment-adjusted prevalence and absolute numbers of positive diagnoses, rather than solely case-finding yield [10,48].

## Scale use of specific testing approaches to reach the untested

### Background

Significant progress on knowledge of HIV status remains variable by region, within countries, and across populations [49]. Adolescent girls and young women have greater access to HTS and ART services due to more frequent engagement with sexual and reproductive health services (especially ANC) compared to adolescent boys and young men. In ESA, only 65% of youth living with HIV (15 to 24 years) know their status (65%), and men aged 35 to 49 years with HIV account for the highest number unaware of their status, with an estimated 701,000 men undiagnosed [2]. Only 66% of children in ESA under 15 years of age know their HIV status compared to 91% of people 15 years and older [1]. Key populations and their partners accounted for 46% of new infections in ESA in 2021 [1] and are a critically undertested population. All of these populations would benefit from improved access to HTS.

### Importance to the future of HIV testing

Despite global guidance recommending implementation of specific evidence-based testing approaches to effectively target and diagnose untested populations [50], investment has been insufficient to scale these approaches. As with facility-based testing, implementation of a mix of community-based models requires adaptation to local needs and resources. Investment should cover commodities and programmatic needs including participation of key populations in HTS implementation. Empowerment of underserved populations requires improved engagement [51]and mobilization of social networks, index cases, and community actors to provide HIV testing, including distribution of HIVST kits [52].

Virtual platforms, index- and social-network testing and other specific population-targeted, community-based testing strategies are likely to increase uptake of testing among key populations, men, and children [53]. Programs need to determine the resources needed to adequately support targeted community-based HTS without depleting facility-based resources.

## Reframe retesting among those previously diagnosed as an opportunity for essential (re)engagement

### Background

It is increasingly recognized that the HIV care continuum is cyclical [54]. Some people with an HIV diagnosis test multiple times, seeking confirmation while delaying (or never) linking to treatment; others retest after disengagement from treatment as a route to re-engagement [55]. Both delayed linkage and disengagement from ART can have negative individual and public health consequences. Among those with HIV who know their status, new data suggest an increasing proportion of people testing positive have previously received an HIV diagnosis [14] For example, in South Africa, half of those testing positive had previously received an HIV diagnosis [56], with only half of them self-reporting prior knowledge of their status.

### Importance to the future of HIV testing

HTS providers may benefit from a reorientation to their vital role in linking and relinking individuals living with HIV to treatment. HTS providers can provide critical support by retesting individuals who are struggling to accept their diagnosis and start ART. In parallel, clients should receive counseling on the benefits of ART for their own health and on the critical value of viral suppression in protecting their sexual partners: increasing knowledge of U = U (undetectable is equal to untransmittable) can reduce self-stigma and anxiety [57]. HTS providers should also accept and enable retesting as a means to facilitate re-engagement in treatment services. Doing so may help facilitate reentry, especially for people who may fear judgmental health providers or who experience a sense of failure associated with not staying on ART [58]. As the epidemic continues to mature, people cycling in and out of care will contribute an increasingly large fraction of transmission [45]. Providing HTS services to those previously diagnosed should be seen as an opportunity, not a burden.

## Involve communities and invest in community-led monitoring

### Background

It is important that people who use HIV services participate in the design, monitoring, and adaptation of services. Increased agency through community-led monitoring (CLM) also supports participation and health system accountability and responsiveness. Effective CLM depends on continuous data collection, analysis, and utilization by clients, health-service providers, and national stakeholders [17].

### Importance to the future of HIV testing

Community participation fosters person-centered services. Communities need to be actively engaged to inform policy, implementation guidance, and demand creation—all of which can be bolstered with better utilization of CLM data. People who use services have important insights into HTS delivery adaptations necessary to reach the unreached. CLM metrics could include HTS quality indicators for both first-time testers and retesters, and for those using HTS to re-engage in prevention and/or treatment services. Increased investment in CLM—financial, technical, and political—can ensure that HTS meet the needs and preferences of people using services.

Details of 4 final themes are outlined in Table 1. In brief, HTS will benefit from being integrated and person-centered with primary healthcare services across a range of health conditions [19]. There are opportunities with virtual interventions and digital tools to bolster the provision of HTS, including but not limited to expanding access to HIVST [24]. The vision of status-neutral testing will benefit from improved HIV health literacy including around U = U messaging [25], and finally, the strategic mix of differentiated HTS will require regular reviews and updates that are data driven.

## Conclusion

Defining a shared vision for the next decade of HIV testing services in ESA is an essential first step to implementing more efficient, effective, and acceptable testing programs that are required to reach and maintain the 2025 UNAIDS prevention and treatment targets. The next step is collaborating with communities, Ministries of Health, health workers, funders, normative agencies, and implementing partners to translate this vision into practice. Successful implementation of this vision will require alignment with global funders and increased autonomy for ministries of health to implement context-driven HTS programming. A shift towards a status-neutral approach to HTS implementation is appropriate in the setting of ESA where “many are transmitting to a few” [45] making linkage and engagement to both prevention and treatment critical. Topics not sufficiently covered by the consultation that need further engagement include how to finance a status-neutral approach to HTS and determining the themes that may be most relevant for other regions. In addition, it is important to recognize that even the countries in ESA are heterogenous; as their epidemics and responses evolve, program data and context-specific studies will be needed to adjust and refine the implementation of the proposed broader, more targeted approach to HTS that will advance both HIV treatment and prevention objectives.

## Supporting information

S1 Appendix Consultation series agenda. (DOCX)

S2 Appendix Participants in *The future of HIV testing*: *Beyond reaching the first 95 in sub-Saharan Africa*” consultation series. (DOCX)

## Abbreviations

ANC: antenatal care
ART: antiretroviral therapy
BMGF: Bill & Melinda Gates Foundation
CLM: community-led monitoring
COVID-19: Coronavirus Disease 2019
ESA: eastern and southern Africa
HIVST: HIV self-testing
HTS: HIV testing service
IAS: International AIDS Society
NSP: needle and syringe programming
OAMT: opioid agonist maintenance therapy
PITC: provider-initiated testing and counseling
PrEP: pre-exposure prophylaxis
STI: sexually transmitted infection
VMMC: voluntary medical male circumcision
WHO: World Health Organization
