# Supplementary material for: The future of HIV testing in eastern and southern Africa: Broader scope, targeted services
Source: PLoS Med. 2023 Mar 14;20(3):e1004182. doi: 10.1371/journal.pmed.1004182 (PMC10013883; doi:10.1371/journal.pmed.1004182)
Supplement: S1 Appendix — (DOCX) [file pmed.1004182.s001.docx]

S1 Appendix:

Consultation series agenda

The future of HIV testing- Beyond reaching the first 95 in sub-Saharan Africa

Session 1: Past success, present priorities, and future opportunities

**Monday, 11 October, 90 mins**, 16:00-17:30 GMT +2

**Agenda**

Session chairs: Rachel Baggaley (WHO) & Florence Anam (GNP+)

|  | Presentation/content | Presenter |
| --- | --- | --- |
| 16:00 – 16:10 | Introduction | Peter Ehrenkranz  (BMGF, USA) |
| 16:10 – 16:20 | Trends in knowledge of HIV status and efficiency of HIV testing services in sub-Saharan Africa | Mathieu Maheu-Giroux  (McGill University, Canada) |
| 16:20 – 16:30 | HIV testing strategies based on current patterns of transmission from HIV phylogenic studies | Christophe Fraser, (Imperial College London, UK) |
| 16:30 – 16:40 | Population-level impact and cost-effectiveness HIV testing strategies for South Africa | Leigh Johnson  (UCT, South Africa) |
| 16:40 – 16:55 | Discussion 1 | Session chairs and discussants |
| 16:55 – 17:05 | The evolution of PEPFAR’s HIV testing strategy – from 2011 to today | Angeli Achrekar  (OGAC, USA) |
| 17:05 – 17:15 | Reflections on client centred testing versus target-based testing: The rationale for maintaining access to and diversification of HIV testing services | Austin Jones  (amfAR, USA) and  Maureen Milanga  (Health GAP, Kenya) |
| 17:15 -17:30 | Discussion 2 | Discussants:  Catherine Ngugi (NASCOP, Kenya), Obinna Onyekwena (Global Fund, Switzerland), others TBC |

Session 2: The different, and sometimes competing, roles of HIV testing

**Monday 25 October, 90 mins**, 16:00-17:30 GMT +2

**Agenda**

Session chairs: Irum Zaidi (S/GAC) & Andreas Jahn (ITECH MALAWI)

|  | Presentation/content | Presenter |
| --- | --- | --- |
| 16:00-16:10 | Introduction to HIV testing for prevention, for treatment and for re-engagement | Cheryl Johnson (WHO, Switzerland) |
| 16:10-16:20 | The importance of HIV testing for prevention – a review of the evidence and successful implementation strategies | Kim Green (PATH, Viet Nam) |
| 16:20-16:30 | The importance of HIV testing for treatment – a review of the evidence and successful implementation strategies | Christian Stillson (CHAI, USA) |
| 16:30-16:45 | Discussion 1 | Session chairs and discussants |
| 16:45-16:55 | The importance of HIV testing for re-engagement – a review of the evidence and successful implementation strategies | Ruanne Barnabas, UW, USA |
| 16:55-17:05 | Lessons from the impact of COVID-19 on HIV testing | Anna Grimsrud (IAS, South Africa) |
| 17:05-17:30 | Discussion 2 | Sessions chairs  Discussants:  Euphemia Sibanda (CESSHAR, Zimbabwe)  Geoffrey Taasi (MoH Uganda), other TBC |

Session 3: Beyond reaching the first 95: the future of HIV testing
Monday 8 November, 90 mins, 15:00-16:30 GMT +1

Agenda

Session chairs: Peter Ehrenkranz (BMGF, USA) and Anna Grimsrud (IAS, South Africa)

|  | Presentation/content | Presenter |
| --- | --- | --- |
| 10 mins | Summary and key takeaways from Session 1 and 2   - Key areas of consensus - Key areas where there is not consensus - Outstanding questions | Helen Ayles, ZAMBART, Zambia |
| 40 mins | Breakout groups – pre-assigned   1. Where there is consensus and we’re doing something right, what is needed to do more of this (operationalize)? 2. What is not resolved or not working? 3. What are the gaps/key areas for consideration that have been missed during this consultation series? | Group 1 facilitators:  Stephanie Behel (CDC, USA) and Tina Chisena (MoH, Zambia)  Group 2 facilitators: Thato Chidarikire, (NDoH, South Africa) and Vincent Wong (USAID, USA) |
| 15 mins | Reflections from the group | Moderated by Cheryl Johnson, WHO, Switzerland |
| 10 mins [2x5] | Where to from here?  Reflections from breakout groups   1. Group 1 report back from one of the facilitators 2. Group 2 report back from one of the facilitators | Group 1 facilitators Group 2 facilitators |
| 15 mins | Next steps from meeting co-convenors | IAS, BMGF, WHO |
