# Supplementary material for: The future of HIV testing in eastern and southern Africa: Broader scope, targeted services
Source: PLoS Med. 2023 Mar 14;20(3):e1004182. doi: 10.1371/journal.pmed.1004182 (PMC10013883; doi:10.1371/journal.pmed.1004182)
Supplement: S2 Appendix — (DOCX) [file pmed.1004182.s002.docx]

S2 Appendix
Participants in *The future of HIV testing: Beyond reaching the first 95 in sub-Saharan Africa*” consultantion series

We acknowledge the contribution of all the participants in the “*The future of HIV testing: Beyond reaching the first 95 in sub-Saharan Africa*”: Angeli Achrekar^1^, George Alemnji^1^, Florence Anam^2^, Helen Ayles^3^, Rachel Baggaley^4^, Amie Baldeh^5^, Solange Baptiste^6^, Taryn Barker^7^, Nelli Barriere^5^, Stephanie Behel^8^, Irene Benech^8^, Chelsea Bond^9^, Laura Broyles^10^, Davina Canagasabey^11^, Gabriel Chamie^12^, Thato Chidarikire^13^, Tina Chisenga^14^, Liz Corbett^15^, Gina Dallabetta^9^, Kathryn Dovel^16^, Jeff Eaton^17^, Peter Ehrenkranz^9^, Ben Eveslage^18^, Christophe Fraser^19^, Damian Fuller^10^, Geoff Garnett^9^, Catherine Godfrey^1^, Matthew Golden^20^, Rachel Golin^1,21^, Lina Golob^5^, Kristina Grabbe^22^, Kimberly Green^11^, Michael Grillo^23^, Anna Grimsrud^5^, Katherine Guerra^24^, Sarah Hamm Rush^9^, Nina Hasen^25^, Karin Hatzold^25^, Brian Honnerman^26^, Amy Huber^27^, Dane Ichimura^9^, Heather Ingold^28^, Andreas Jahn^29^, Cheryl Case Johnson^4^, Leigh Jonson^30^, Austin Jones^26^, Sara Klucking^1^, Catey Laube^22^, Susan Lorente^31^, Mathieu Maheu-Giroux^32^, Mary Mahy^33^, David Maman^31^, Susie McLean^31^, Sajay Menon^9^, Gesine Meyer-Rath^27^, Maureen Milanga^34^, Khumbo Namachapa^35^, Getrude Ncube^36^, Emi Okamoto^24^, Obinna Onyekwena^31^, Kaitlin Powers^21^, Josie Presley^9^, Miriam Rabkin^37^, Tanya Shewchuk^9^, Euphemia Sibanda^38^, Aayush Solanki, Christian Stillson^24^, Andrew Storey^24^, Jessica Sun^9^, Geoffrey Taasi^39^, Melissa Wilde^9^, Lynne Wilkinson^5^, Emma Williams^10^, Vincent Wong^21^, Sinokuthemba Xaba^36^, Nicole Young^9^, Irum Zaidi^1^

^1^ Office of the Global AIDS Coordinator, USA, ^2^ GNP+, Kenya, ^3^ ZAMBART, Zambia, ^4^ World Health Organization, Switzerland, ^5^ IAS – the International AIDS Society, Switzerland, ^6^ International Treatment Preparedness, South Africa, ^7^ Children's Investment Fund Foundation, UK, ^8^ Centers for Disease Control and Prevention, USA, ^9^ Bill & Melinda Gates Foundation, USA, ^10^ Eureka Idea Co, ^11^ PATH, USA, ^12^ University of California San Francisco, USA, ^13^ National Department of Health, South Africa, ^14^ Ministry of Health, Zambia, ^15^ London School of Hygiene and Tropical Medicine, UK, ^16^ University of California Los Angeles, USA, ^17^ Imperial College London, UK, ^18^ FHI 360, USA, ^19^ University of Oxford, UK, ^20^ University of Washington, USA, ^21^ United States Agency for International Development, USA, ^22^ Jhpiego, USA, ^23^ Department of Defence, USA, ^24^ Clinton Health Access Initiative, USA, ^25^ PSI, USA, ^26^ Foundation for AIDS Research, USA, ^27^ University of Witwatersrand, South Africa, ^28^ Unitaid, Switzerland, ^29^ I-TECH, Malawi, ^30^ University of Cape Town, South Africa, ^31^ The Global Fund, Switzerland, ^32^ McGill University, Canada, ^33^ Joint United Nations Programme on HIV and AIDS, Switzerland, ^34^ Health GAP, Kenya, ^35^ Ministry of Health, Malawi, ^36^ Ministry of Health and Child Care, Zimbabwe ^37^ ICAP at Columbia University, USA, ^38^ Centre for Sexual Health and HIV/AIDS Research, Zimbabwe, ^39^ Ministry of Health, Uganda
